# Timely recognition of a probably life-threatening genodermatosis: familial case report of hereditary leiomyomatosis and renal cell cancer

**DOI:** 10.3389/pore.2025.1612086

**Published:** 2025-04-08

**Authors:** Judit Kárteszi, Nikoletta Nagy, Márta Széll, Zsuzsanna Lengyel, Dávid Semjén, Zsolt Egyházi, Gábor Bajzik, Levente Kuthi, Csaba Pusztai, Zita Battyáni

**Affiliations:** ^1^ Genetic Counselling, Saint Raphael Hospital of Zala Castle County, Zalaegerszeg, Hungary; ^2^ Institute of Medical Genetics, University of Szeged, Szeged, Hungary; ^3^ Department of Dermatology, Venereology and Oncodermatology, University of Pécs, Pécs, Hungary; ^4^ Department of Pathology II. Histology and Cytology, University of Pécs, Pécs, Hungary; ^5^ Department of Pathology, Somogy County Moritz Kaposi Teaching Hospital, Kaposvár, Hungary; ^6^ Dr. Baka József Diagnostic, Radiation Oncology, Research and Teaching Center, Somogy County Moritz Kaposi Teaching Hospital, Kaposvár, Hungary; ^7^ Department of Surgical and Molecular Pathology, Tumor Pathology Center, National Institute of Oncology, Budapest, Hungary; ^8^ Department of Pathology and Experimental Cancer Research, Semmelweis University, Budapest, Hungary; ^9^ Department of Urology, Somogy County Moritz Kaposi Teaching Hospital, Kaposvár, Hungary; ^10^ Department of Dermatology, Somogy County Moritz Kaposi Teaching Hospital, Kaposvár, Hungary

**Keywords:** fumarate hydratase deficiency, renal cell cancer, hereditary leiomyomatosis, autosomal dominant, case report

## Abstract

**Background:**

Autosomal dominant genodermatoses with a predisposition for cancer make up a well-described disease group with unique cutaneous alterations in each. This should urge dermatologists to think of other consequences beyond the skin. Histological examination serves as the gold standard, and it is an effective tool for the first investigation, even nowadays in the “next-generation genetic” era. Multiple appearances of benign tumours histologically proved to be cutaneous leiomyomatosis suggest a rare disorder with germline heterozygous pathogen variant in the *FH* gene. The encoded fumarate hydratase is a Krebs cycle enzyme, and has a role in catalysing the transition from fumarate to malate.

**Case presentation:**

Years before the easy accessibility of the complete genetic workup in Hungary, a yearly abdominal MRI check-up was suggested preventively for a middle-aged man with multiplex cutaneous leiomyomata. During the follow-up period papillary type 2 renal cell carcinoma was diagnosed in the left kidney at an early stage, and a successful operation saved his life without the need for aggressive chemotherapy or immunotherapy. Immunohistochemistry of tumour tissue proved FH-deficient renal cell cancer. We discuss in short the current knowledge of pathophysiology and accessible therapies regarding this aggressive malignant tumour type in the kidney, which is usually detected in the advanced stage with early metastasis. We also highlight an early sign, i.e., solitary cystic alteration in the kidney, which can be preliminarily observed before malignant transformation, which was also described in mouse models. Sanger sequencing and Multiplex-Ligation-Dependent Probe Amplification (MLPA) analysis of the *FH* gene was completed in the affected son of the original proband, and Hereditary Leiomyomatosis and Renal Cell Cancer (HLRCC) was confirmed by demonstrating a large germline deletion in this family after years of observation.

**Conclusion:**

Regular observation of individuals with hereditary leiomyomatosis may prevent a serious sequelae of untreatable renal malignancy.

## Introduction

Plenty of genodermatoses that are inherited autosomal dominantly can predispose the bearer to cancer, which means that early detection and correct diagnosis must be a requirement [1]. Within this broad group of diseases, special genetic disorders belong to the hereditary renal cancer syndromes [2, 3].

Samples of eleven autosomal dominant families segregating multiple leiomyomatosis were used for genome-wide screening, which found evidence of a linkage to chromosome region 1q42.3-q43, and the minimal interval containing the locus was determined in 2001. A tumor suppressor gene called *MCUL1* (Multiple Cutaneous and Uterine Leiomyomata 1) was determined in the background at that time. Based on the current nomenclature, autosomal dominant Hereditary Leiomyomatosis and Renal Cell Cancer (HLRCC, MIM150800) describes the exact cancer syndrome with germline heterozygous variant of the *FH* gene, which encodes fumarate hydratase, an enzyme in the Krebs cycle [4]. The essential role of fumarate hydratase can be supported by the existence of autosomal recessive Fumarate hydratase deficiency, which is an inborn error of metabolism with severe consequences from birth, including encephalopathy, failure to thrive and severe neurological deficit.

The clinical picture of HLRCC can be characterized by skin leiomyomas, uterine fibroids and type 2 papillary kidney cancer in a significant portion of patients [5, 6]. Data from the literature has confirmed incomplete penetrance, which increases with age. The expressivity may be different in various members of the same family, and quality of life may be severely lowered in some of the affected individuals due to intense pain and a torsion effect on some skin lesions. Unusual unilateral distribution of leiomyomas is possible, and a large number of lesions can appear (more than a hundred). The appearance of the first piloleiomyomas (PLM) is in adolescence or early adulthood. These benign skin tumours originate in the arrector pili muscles of the hair follicles and grow in number and size. PLMs have characteristic histologic features such as poorly defined outlines, entrapped hair follicles and eccrine glands, acanthosis and elongated rete ridges with hyperpigmentation and smooth muscle bundles, which are interdigitated with elongated rete ridges [7]. For diagnosis, the only major criterium is the appearance of more than one histologically proved PLM, and the minor criteria include operations for multiple uterine leiomyomas and type 2 papillary renal cell carcinoma, both before the age of 40 years, and having a first-degree relative who also fulfils these criteria. The diagnosis of HLRCC should be strongly considered if a patient displays either the one major diagnostic criteria or two of the three minor criteria. Pain in the lesions can be provoked by cold or pressure. Some authors suggest that aetiology of the pain is the pressure on the nerves within the lesions, while others indicate that it is muscle contractions mediated via alpha-adrenergic receptors. Germline mutations in *FH* predispose women to early onset and multiple uterine leiomyomata [8]. HLRCC-associated renal cell cancer (RCC) was recognized as a separate category in the World Health Organization (WHO) classification of renal tumours in 2016. A patient diagnosed with HLRCC has 25–30 percent chance of having an aggressive malignant renal tumour. HLRCC-associated RCC stands in the focus of intensive research not only in connection with basic molecular processes [9–11], but also in developing new therapeutic approaches [12].

We present two individuals, a father and his son, who experienced the appearance of extensive cutaneous leiomyomata in young adulthood and who were primarily examined at the local Department of Dermatology. Originally the father was examined, and HLRCC was suggested based on clinical criteria [13] because *FH* gene analysis could not be fully performed at that time. However, thanks to the advice of yearly MRI scanning of the kidneys, aggressive type 2 renal carcinoma of papillary was diagnosed in an early stage, and immunohistochemistry proved fumarate hydratase deficiency in the renal tumour. The workup of the son’s DNA sample was possible some years later with Sanger sequencing and Multiplex-Ligation-Dependent Probe Amplification (MLPA) method, so subsequently, genetic diagnosis could be determined in this family.

## Case description

### Individual I

**FIGURE 1:**
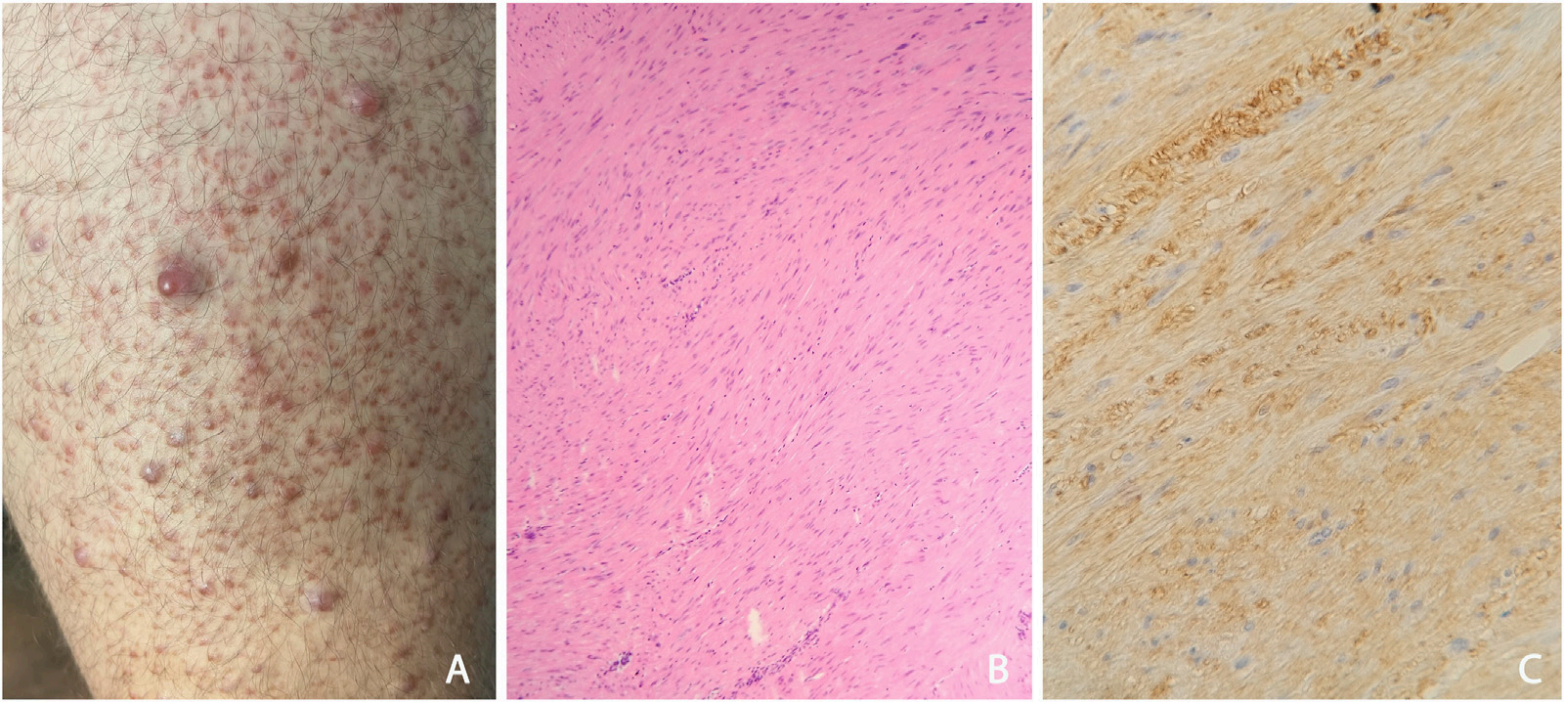
**(A)** Appearances of typical cutaneous leiomyomas: multiple red nodules, surrounded by follicular red papules at the anterior surface the left thigh **(B)** Histological study of the benign skin tumour: dermal disorganised, elongated, leiomyomatous bundles; HE, 50x **(C)** In the dermal tumour, leiomyoma cells express smooth muscle actin in immunohistochemistry; SMA IHC, 100x.

This male patient is currently 30 years old. He first visited our department at age of 18 due to the appearance of painful skin nodules. His skin lesions were located mainly on his lower extremities: a large number of 1-2 mm hyperaemic papules were observed attached to follicles (Figure 1A), and some 1 cm large nodules were found separately, too. Histological examination verified cutaneous leiomyomatosis, composed by dermal smooth muscle fibres with irregular structure expressing smooth muscle actin in immunohistochemistry (Figures 1B, C).

Symptoms correlated with painful progressive growth of cutaneous nodules, which were aggravated by low temperatures. Non-steroid analgesics were not efficacious. The benign lesions were located symmetrically on both sides of the trunk, and the original tumours on the thighs enlarged to 1.5 cm in diameter. Local surgical therapy of the largest lesions was suggested but the patient accepted neither surgery nor laser removal. The patient’s clinical history was otherwise negative, and annual MRI controls had not detected any malignancies in the kidneys. Due to the dispersed appearance and pain progression in cold weather, we introduced gabapentin treatment.

Entire sequencing analysis of the *FH* gene was performed in this individual with Sanger sequencing, and no pathogenic variant was detected (DNA was isolated from peripheral blood). The MLPA technique was used to investigate the possibility of large deletions in the *FH* gene. From the MLPA results, a heterozygous pathogenic deletion of the *FH* gene was identified. His wife is currently pregnant, and a 50 percent recurrence risk was communicated at genetic counselling.

### Individual II

A 51 year-old man, who is the father of Individual I. He had complaints from the age of 14, when painful and constantly but slowly growing red papules appeared, first on his extremities and later on his trunk. At age 33, histological analysis proved the diagnosis of pilar leiomyomatosis in the background. Later, we observed the patient when he was 44 years old, and we found scattered benign tumours that were 3–7 mm in diameter on his face, trunk and extremities. These were slightly painful when pressure was applied.

His family history was suggestive of autosomal dominant inheritance. His father, paternal grandmother and adolescent son had similar skin lesions, but kidney cancer was not noted in family history. Uterine myomatosis was not observed in female individuals in the family. The diagnosis of Hereditary Leiomyomatosis Renal Cell Cancer (HLRCC) was suspected. However, at that time it was possible only to perform histological analysis.

**FIGURE 2:**
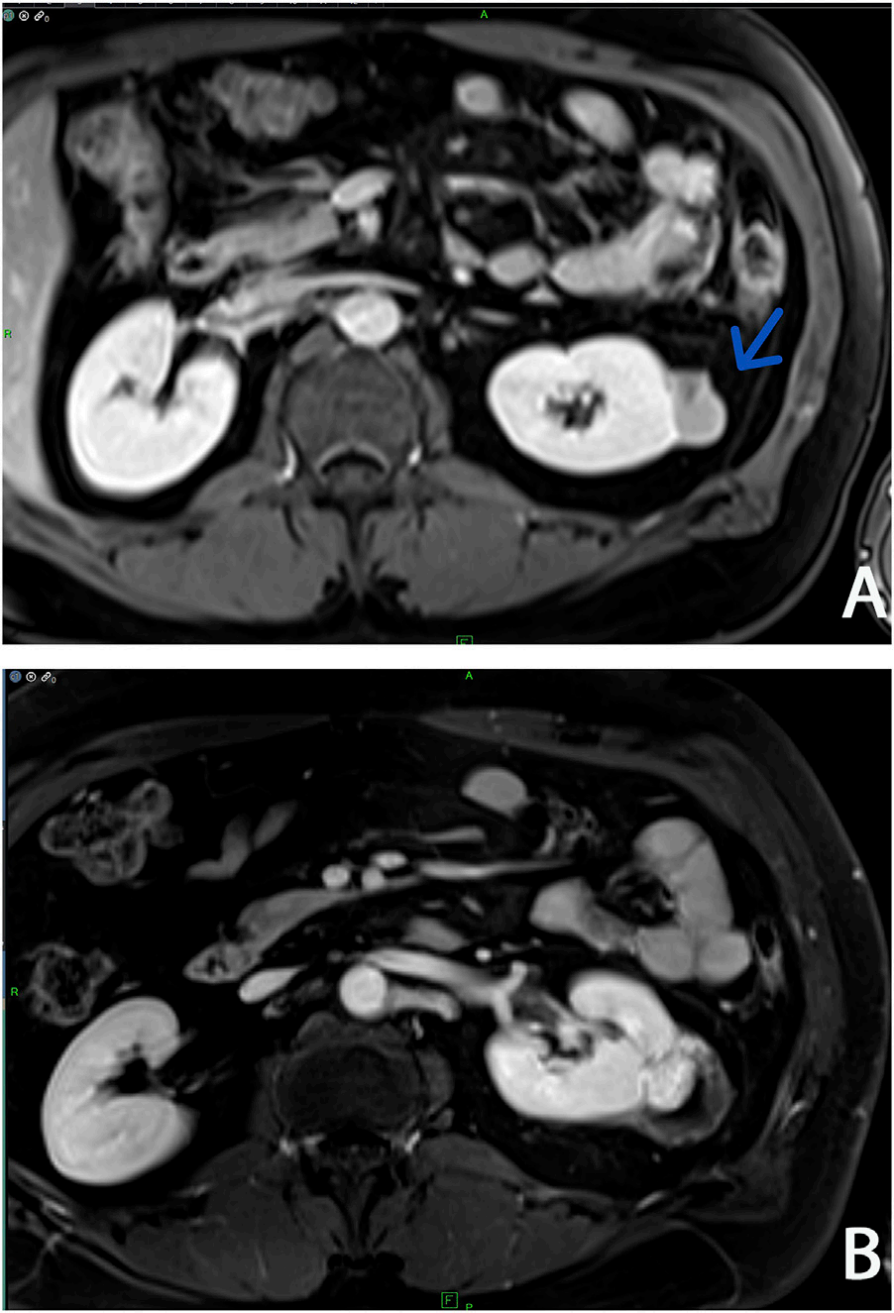
Abdominal contrast-enhanced T1-weighted MRI imagings at two different times: **(A)** Arrow shows a localized complex cystic lesion (Bosniak III, cyst) on the left kidney **(B)** Three years later an increasing cystic lesion with hyperenhanced solid component (Bosniak IV, cystic tumor) was detected on the same side.

**FIGURE 3:**
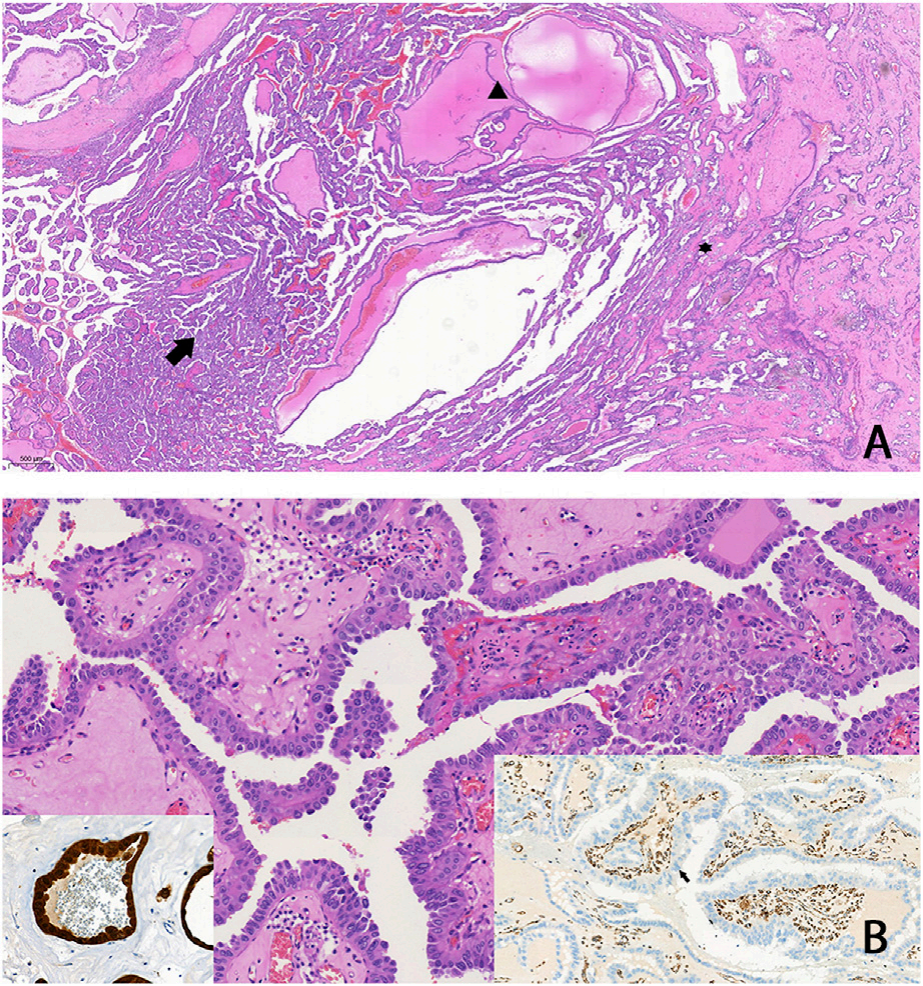
FH-deficient RCC: **(A)** A tumour that predominantly exhibits papillary structures (arrow), alongside cystic (arrowhead) and tubular formations (asterisk). Typically, high-grade cytomorphology is observed, characterized by abundant eosinophilic (oncocytic) cytoplasm, which made the classification of these tumours challenging in the past **(B)** Negative immunohistochemical staining for FH in tumour cells (arrow on right insert), and/or aberrant staining for 2SC (left insert) is highly sensitive for FH-deficient RCC.

**FIGURE 4:**
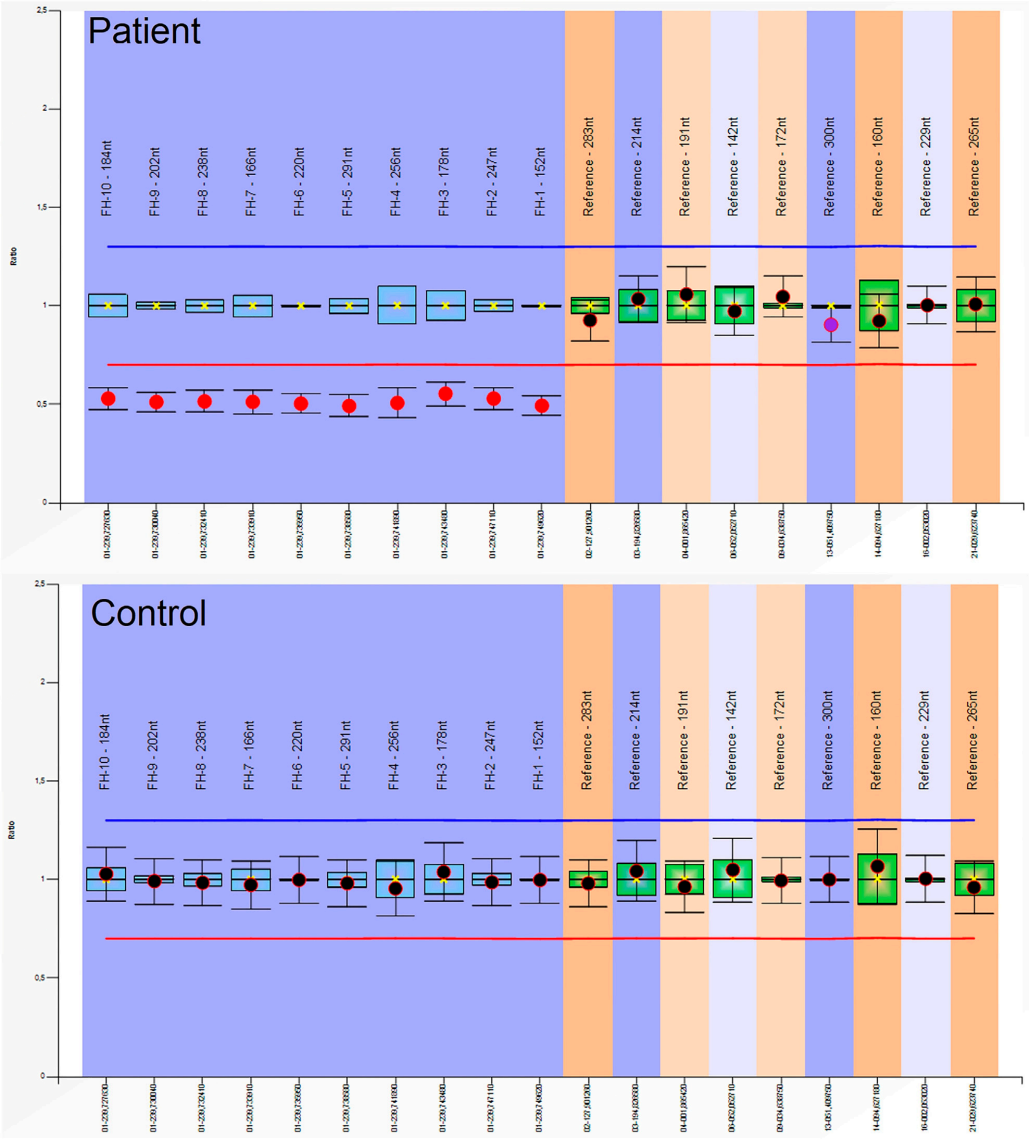
Multiplex-Ligation-Dependent Probe Amplification (MLPA) analysis of the *FH* gene in the patient and in a healthy control: A heterozygous pathogenic deletion of the *FH* gene was identified in the patient. We used SALSA MLPA Probemix (MRC-Holland, Netherlands) containing probes for the *FH* gene, according to the manufacturer’s instructions. Amplicon fragment length analysis was performed on an ABI 3500 Genetic Analyzer (ThermoFisher Scientific, Waltham, MA, United States) and analysed using Coffalyser.net software (MRC-Holland, Amsterdam, Netherlands).

Abdominal contrast-enhanced MRI was indicated after genetic consultation, to exclude the possibility of association with a malignant kidney tumour. This revealed a 27 × 19 × 15 mm large atypical cystic lesion in the left kidney (Figure 2A), without contrast material uptake, so malignant tumour was not detected. Regular check-ups were recommended for early detection in case of malignant disease. He also had regular urology check-ups because of complaints of urolithiasis. At 47 years of age, before the yearly abdominal check-up, he felt severe pain in his left lower back region after lifting heavy weights. An ultrasound suggested subcapsular bleeding, but during a later MRI it was also observed that the cyst that had been detected previously had grown to 40 × 35 × 35 mm (Figure 2B). Solid parts with contrast enhancing and the multi-compartment cystic surrounding suggested kidney cancer. Laparoscopic tumour resection was performed. The histological examination found a crusted, pseudo capsular lined cystic renal cell carcinoma with anisocytosis and anisonucleosis. Inclusions and nuclear grooves were detected in the cell nucleus. Histological diagnosis was type 2 renal cell papillary carcinoma, which was in correlation with the clinical diagnosis of HLRCC. The tumour mass was completely removed and chemotherapy was not indicated. After 7 years of follow-ups he is living without tumour recurrence. Subsequently, further immunohistochemistry was performed, which proved tumour cells with fumarate hydratase deficiency (Figure 3). A large germline *FH* gene deletion was proved by MLPA analysis (Figure 4) years after the successful surgery for the malignant kidney cancer.

## Discussion

Hope for a real curative method for a genetic disorder in the future can be based only on a proper understanding of pathophysiology. The last few decades of basic research into cancer is a good example of this, and has led to several types of biological therapies becoming available. At this time several previously completely untreatable conditions now have some type of treatment, and enormous development has been witnessed in the field of RCC [14]. Most tumours are asymptomatic at incidental detection, which was the case also in Individual B. However, later diagnosing HLRCC associated RCC may unravel an aggressive mass with early metastasis. It is characteristic that this type of carcinoma tends to be more infiltrative and not well circumscribed if diagnosed too late. HLRCC patients usually have earlier onset of their renal tumor. As Sanger sequencing didn’t identify the pathogenic germline variant of the *FH* gene in the MLPA-positive patient, our results highlight the importance of the combination of these screening methods for the genetic diagnosis of the disease.

The basic alteration of hereditary leiomyomatosis is the germline heterozygous variant of the *FH* gene [15], and loss-of-heterozygosity at this locus cause the complete loss of enzyme function of fumarate hydratase in the tumor tissue. This loss may be demonstrated in cutaneous leiomyomata with immunohistochemistry suggesting the diagnosis as early as possible. The highly conserved sequence of *FH* dedicate the important role of this enzyme in the Krebs cycle, which is the essential route to oxidative phosphorylation the aerobic and effective process for energy production. FH deficiency causes dependence on glycolysis and drives special pathway alterations in the diseased cells (for example DNA damage response pathway). Fumarate hydratase has the function to catalyse the conversion of fumarate to malate, and lack of FH activity leads to fumarate excess in the cell. Fumarate excess causes posttranslational modification on cysteine residues called succination [16], which leads to 2-succinyl cysteine on targeted proteins (immunohistochemistry staining of the overexpression of 2-succinyl cysteine can discriminate HLRCC from sporadic tumours). The succination alters cellular iron homeostasis (ferritin elevates, free iron lowers) in HLRCC and induces a pro-proliferative signalling. The main target protein for succination is KEAP1, which is known to impact the regulation of the NRF2-KEAP1 pathway. The altered proteins take part in redox signalling. The overactivation of the transcription factor named NRF2 was determined in the background of HLRCC [9–11].

It has been proven that fumarate hydratase belongs to the same pathway as VHL (Von Hippel-Lindau), which is a key-substrate adapter for an important ubiquitin ligase complex, which mediates the ubiquitylation of the hydroxylated forms of HIF1A and HIF2A (Hypoxia Inducible Factors). Without this process these factors become constitutively activated and cause pseudohypoxia and Von Hippel-Lindau disease. The impaired function of another enzyme in the Krebs cycle, called succinate dehydrogenase, also leads to pseudohypoxia, because of the accumulation of succinate, and the causative inhibition of propyl hydroxylase, the enzyme which hydroxylate HIF1A and HIF2A (the name of the associated tumor syndrome is Hereditary Pheochromocytoma and/or Paraganglioma). Similarly, an excess of fumarate has been hypothesised to inhibit propyl hydroxylase and cause constitutive HIF activation.

The knock-out mouse model for FH deficiency in the kidney caused the development of several cysts which were premalignant hyperproliferative lesions. We observed a slowly growing cystic lesion in the kidney on an MRI performed in our patient years before the malignant transformation. At this time we can state that early detection and surgical excision of the tumour is the most effective treatment. Currently all systemic therapies in advanced cases have limited efficacy. We summarize therapeutic possibilities based on the knowledge of the molecular sequalae caused by fumarate hydratase deficiency in tumour tissue detailed above. As only the tumor cells harbour biallelic *FH* variant, it is a good candidate for targeted therapy (Supplementary Table S1).

There are alternative options for local therapy for skin lesions, although these may be difficult and inadequate. There have not been any wide-ranging studies concerning these types of treatments, and only some published cases present successful effects. The treatment depends on the clinical appearances of the disease. In cases with solitary or moderately scattered tumours, either local surgical excision or other locally destructive treatments as CO2- and ErbiumYAG-laser or cryotherapy [17–19] are suggested. Local triamcinolon acetonid infiltration is also useful [20]. In the disseminated cases systemic treatment is recommended. In cases with muscle contraction-based pain, an alfa adrenergic receptor inhibitor (doxasosine) can be used, which resolves the muscle spasms and leads to a pain reduction [21]. The Ca channel inhibitors nifedipine and amlodipine are widely used and are effective [22, 23]. There are several publications about the positive effect that gabapentin and pregabalin have on pain [24, 25]. Oral Nifedipine, nitroglycerin, fenoxibenzamin, ß blocker and doxazosin had varying degrees of success in reducing pain. Local injections of triamcinolone acetonide weekly for 3 weeks in the nodules relieved pain and alterations became smaller. The use of the toxin botulinum provided a decrease in the amount of analgesics [26]. In addition to the listed therapeutic options, numerous side effects are well known, which lead to common changes in the therapy applied. The main goal is complete pain relief, because this disorder often leads the patient to take pain killers regularly.

## Conclusion

Our case presentation emphasizes the need for a thorough follow up of patients with a possible genetic disease that predisposes them to cancer in order to prevent a currently untreatable malignancy.

## Data Availability

The raw data supporting the conclusions of this article will be made available by the authors, without undue reservation.

## Abbreviations

HLRCC, Herediter leiomyomatosis and renal cell cancer; FH, fumarate hydratase; MCUL1, Multiple Cutaneous and Uterine Leiomyomata 1; PLM, piloleiomyomas; WHO, World Health Organization; DNA, deoxyribonucleic acid; RCC, renal cell cancer.
