# Supplementary material for: Timely recognition of a probably life-threatening genodermatosis: familial case report of hereditary leiomyomatosis and renal cell cancer
Source: Pathol Oncol Res. 2025 Apr 8;31:1612086. doi: 10.3389/pore.2025.1612086 (PMC12011599; doi:10.3389/pore.2025.1612086)
Supplement: Supplementary file 1 [file DataSheet1.PDF]

Table 1. Possible therapeutic targets in HLRCC-associated RCC

CONNECTION BETWEEN PATHOPHYSIOLOGY AND THERAPY
A PROTEOSOME INHIBITOR BORTEZOMIB ELEVATES REACTIVE OXYGEN SPECIES LEVEL AND LEADS TO APOPTOSIS.
CONSTANT NRF2 ACTIVATION IS DEPENDENT ON GLUTAMINOLYSIS, SO INHIBITION OF GLUTAMINASE MAY SELECTIVELY KILL CANCER CELLS.
GLYCOLYSIS BLOCKING THERAPIES I.E. LACTATE DEHYDROGENASE A INHIBITION PRESENT IN FOCUS OF FURTHER RESEARCH.
FH INACTIVATION SENSITIZES CELLS TO FERROPTOSIS, AN IRON-DEPENDENT CELL-DEATH MECHANISM IS AN OPTION.
HE SUCCINATED FORM OF GPX4 HAS LOWERED ACTIVITY. CYSTEINASE THERAPY IS IN A PRECLINICAL STAGE.
ADENYLATE CYCLASE WAS IDENTIFIED AS THE SYNTHETIC LETHAL TARGET OF FH-INACTIVATED CELLS.
THERAPEUTIC TARGET SCREENING METHODS WERE DEVELOPED EITHER WITH SHORT HAIRPIN RNAS OR THE CRISPR TECHNOLOGY. SHRNA SCREEN FOUND PHOSPHOGLUCONATE DEHYDROGENASE (PGD) KNOCKDOWN A LETHAL COMBINATION WITH FH INACTIVATION. PGD INHIBITION BLOCKS GLYCOLYSIS, BUT MORE SPECIFIED PGD INHIBITORS ARE NEEDED TO AVOID NEUROTOXICITY.
